# 2294 Do patient comorbidities impact the effectiveness of a COPD self-management program?

**DOI:** 10.1017/cts.2018.163

**Published:** 2018-11-21

**Authors:** Emilia Galli Thurber, Hanan Aboumatar

**Affiliations:** Johns Hopkins University School of Medicine

## Abstract

OBJECTIVES/SPECIFIC AIMS: Chronic obstructive pulmonary disease (COPD) is a leading cause of both hospitalizations and readmissions in the United States, and about 1 in 5 hospitalized patients with COPD will be readmitted within 30 days. COPD-focused self-management programs are frequently used to help patients better manage their symptoms and prevent hospitalization. However, while the majority of patients with COPD have at least one comorbidity, most trials of COPD self-management programs either excluded patients with significant comorbidities or did not analyze the impact of comorbidities on patient outcomes. Using data from the BREATHE trial of a COPD self-management program, this study aims to determine if patient post-intervention outcomes differ based on the intensity and type of patient comorbidities. METHODS/STUDY POPULATION: In total, 240 patients hospitalized for COPD were randomly assigned to either a comprehensive self-management intervention or usual transitional care. Primary outcomes for this trial were the number of COPD-related hospitalizations and emergency department visits at 6 months and changes in COPD-specific quality of life. To determine whether patient comorbidities modify the effect of the self-management intervention on readmission and quality of life outcomes, we will compare patient outcomes across groups stratified by comorbidity burden (Charlson Comorbidity Index) and type (baseline diagnosis of congestive heart failure, diabetes, and depression). In addition, we will use regression analysis with interaction terms to test for interaction between comorbidity burden/type and intervention assignment. RESULTS/ANTICIPATED RESULTS: We hypothesize that the effect of the self-management intervention will differ in patients with greater comorbidity burden due to competing medical demands for patients with multimorbidity. DISCUSSION/SIGNIFICANCE OF IMPACT: The results of this study will help clinicians better target disease-specific self-management programs to the groups of patients with COPD who are likely to receive the greatest benefit from this type of intervention.

